# Multidisciplinary Management of Severe Maternal H1N1 Infection With Acute Respiratory Distress Syndrome and Ventilator-Associated Pneumonia Requiring Extracorporeal Membrane Oxygenation: A Case Report

**DOI:** 10.7759/cureus.93129

**Published:** 2025-09-24

**Authors:** Ashlin Z Thomas, Arjun Sreekumar, Ashtami Krishnan, Gopika Balachandran, Philip Mathew

**Affiliations:** 1 Department of Critical Care Medicine, Believers Church Medical College Hospital, Thiruvalla, IND; 2 Department of Cardiothoracic Surgery, Dr. K. M. Cherian Institute of Medical Sciences, Kallissery, IND

**Keywords:** ecmo, maternal h1n1, multi-disciplinary teams, severe respiratory distress syndrome, ventilator associated pneumonia

## Abstract

Pregnant women are at increased risk of severe H1N1 infection, which can lead to complications such as acute respiratory distress syndrome (ARDS) and secondary infections. We report a 29-year-old woman at 29 weeks of gestation who developed fever, cough, and rapidly worsening respiratory distress. Initial management included oxygen therapy, Bilevel Positive Airway Pressure (BiPAP), antivirals, antibiotics, and antenatal corticosteroids; however, worsening hypoxemia necessitated an emergency cesarean section, resulting in the delivery of a viable preterm infant. Postoperatively, she required mechanical ventilation, prone ventilation, and venovenous extracorporeal membrane oxygenation (ECMO). Ventilator-associated pneumonia (VAP) due to multidrug-resistant organisms was successfully treated with targeted antibiotics. The patient was gradually weaned from ECMO and ventilatory support and discharged in stable condition with her newborn after 40 days. This case highlights the importance of timely obstetric intervention, advanced respiratory support, and multidisciplinary coordination in achieving favorable maternal and neonatal outcomes, and demonstrates successful maternal survival with ECMO and treatment of multidrug-resistant VAP during pregnancy.

## Introduction

H1N1 infection, commonly known as swine flu, is caused by the H1N1 virus, a subtype of influenza A. The clinical spectrum ranges from mild upper respiratory symptoms, including rhinorrhea, cough, myalgia, headache, and fever, to severe complications such as viral pneumonia, acute respiratory distress syndrome (ARDS), and secondary bacterial sepsis [1].

Pregnancy is associated with suppressed innate immunity, which delays initial viral clearance and increases influenza severity, placing pregnant women at higher risk of hospitalization and death [2]. Influenza infection during pregnancy, particularly with severe or pandemic strains like H1N1, also substantially elevates the risk of adverse fetal outcomes, including stillbirth, highlighting the need for early recognition and aggressive multidisciplinary management to optimize both maternal and fetal survival [3].

Extracorporeal membrane oxygenation (ECMO) has demonstrated significant benefits in patients with severe respiratory insufficiency, promoting early recovery and favorable outcomes [4]. Timely delivery may be life-saving for both mother and fetus, helping to improve maternal respiratory mechanics and fetal outcomes. We present the case of a previously healthy 29-year-old woman at 29 weeks of gestation, illustrating the critical role of early, specialized care and timely delivery in managing ARDS during pregnancy.

## Case presentation

**Figure 1:**
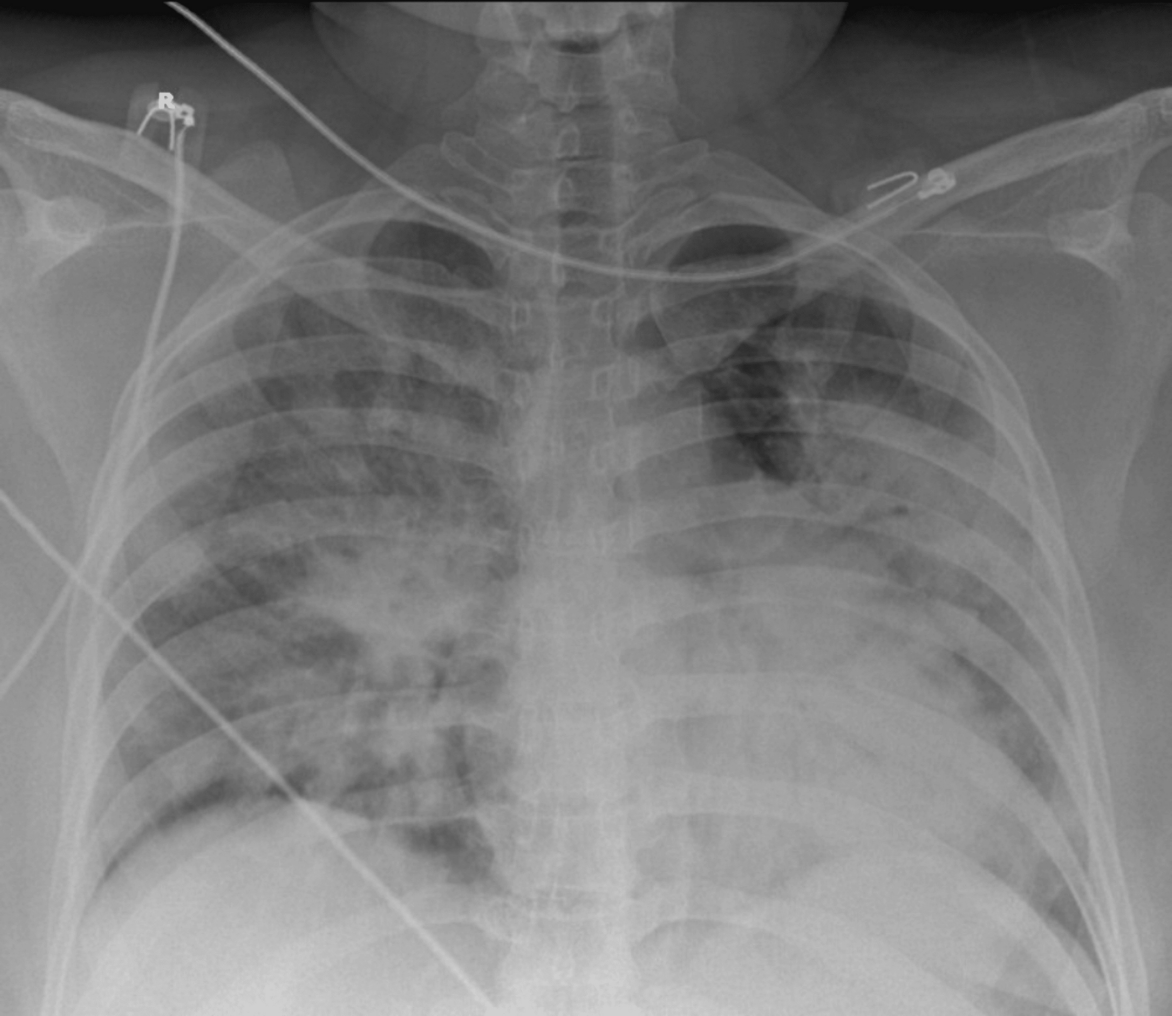
Chest X-ray showing diffuse parenchymal infiltrates consistent with ARDS. ARDS, acute respiratory distress syndrome

A 29-year-old woman, gravida 2 para 1, with a prior first-trimester abortion, was admitted with symptoms of body ache, fever, cough, and respiratory distress. She was 29 weeks and 1 day along in her pregnancy with regular antenatal visits and scans, which revealed a healthy intrauterine fetal growth and no obvious maternal or fetal abnormalities. She was referred to a tertiary center in view of increasing respiratory distress. Upon arrival, she was tachycardic (126 beats/minute), tachypneic (40 breaths/minute), febrile at 38°C, hypoxemic, maintaining an SpO₂ of 85% on 10 L/minute via face mask. On auscultation, bilateral basal crepitations were heard. She was rapidly switched to bilevel positive airway pressure (BiPAP) (fraction of inspired oxygen (FiO₂), 100%, pressure support (PS), 16 cmH₂O, and positive end-expiratory pressure (PEEP), 5 cmH₂O), following which no improvement was seen. Arterial blood gases (ABG) obtained revealed severe hypoxia with PaO₂ of 41.5 mmHg, PaCO₂ of 24.2 mmHg, HCO3- of 16.5 mmol/L, and elevated lactate of 3.1 mmol/L. A subsequent chest X-ray revealed diffuse parenchymal infiltrates consistent with ARDS (Figure 1).

To rule out pulmonary embolism in the setting of an elevated D-dimer (6,278 ng/mL), bilateral lower limb Doppler ultrasound revealed no evidence of deep vein thrombosis. Nasal swab PCR testing was positive for H1N1, Category C according to the categorization used by Indian health authorities to identify severe cases requiring hospitalization and antiviral therapy [5]. Fetal assessment at presentation showed a normal cardiotocography with a heart rate of 151 bpm. Obstetric doppler ultrasound demonstrated normal umbilical artery, middle cerebral artery, and uterine artery flow, with no evidence of fetal growth restriction or placental insufficiency. Considering the high risk of preterm delivery, intramuscular betamethasone 12 mg, with a plan to repeat after 24 hours, was administered to promote fetal lung maturity. She was also started on oseltamivir, intravenous meropenem, and oral azithromycin, in view of an elevated total leukocyte count of 18,700/μL, CRP of 105.9 mg/ L, and procalcitonin of 5.48 ng/mL. Despite these measures, her oxygen requirements escalated, necessitating ICU transfer. On admission, the Acute Physiology and Chronic Health Evaluation II (APACHE II) score was 14, corresponding to an estimated mortality of 15%. A prompt multidisciplinary team discussion involving intensive care, obstetrics, neonatology, pulmonology, internal medicine, and cardiothoracic and vascular surgery (CTVS) guided further decision-making.

Due to worsening maternal respiratory distress and potential fetal compromise, an emergency lower-segment cesarean section (LSCS) was performed the same day. The patient with American Society of Anesthesiologists (ASA) grade III was placed under general anesthesia using rapid sequence induction in a semi-propped position of 30°-40°. Intraoperatively, meconium-stained amniotic fluid was noted, and the placenta and membranes were delivered intact, which appeared grossly normal. Bilateral fallopian tubes and ovaries were unremarkable. A live preterm female infant weighing 1.27 kg was delivered in cephalic presentation with Apgar scores of 6 and 8 at 1 and 5 minutes, respectively. No immediate neonatal complications were observed. The umbilical cord was promptly clamped and cut, and the newborn was transferred to the NICU for monitoring.

**Figure 2:**
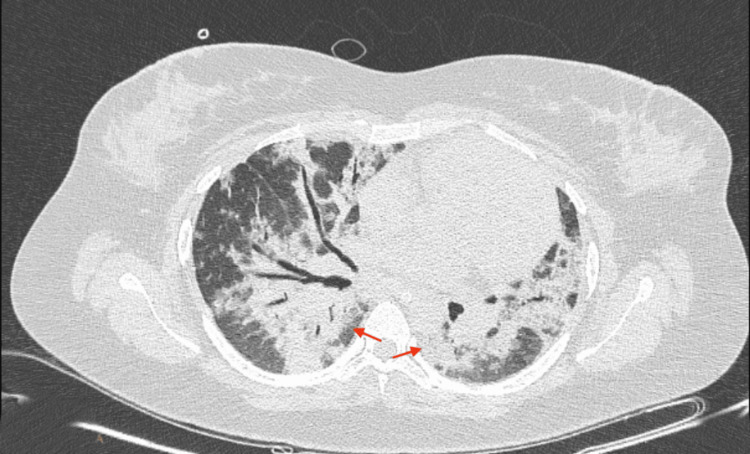
CT scan of the chest showing bilateral ground-glass opacities, consolidation, and interstitial thickening (arrows).

Postoperatively, the patient was hemodynamically stable on mechanical ventilation and shifted to the ICU. The patient had adequate, clear urine output and no significant immediate postoperative bleeding. However, due to persistently high oxygen demand, she required sedation and paralysis with pressure control ventilation (FiO₂ 100%, PEEP 15 cmH₂O) and had a PaO₂/FiO₂ ratio of 85. The patient underwent a CT scan of her chest, which revealed bilateral ground-glass opacities, consolidation, and interstitial thickening (Figure 2).

She then underwent prone ventilation for 12 hours following LSCS. However, serial ABGs showed no significant improvement in oxygenation, with the PaO₂/FiO₂ ratio remaining at 89.

**Figure 3:**
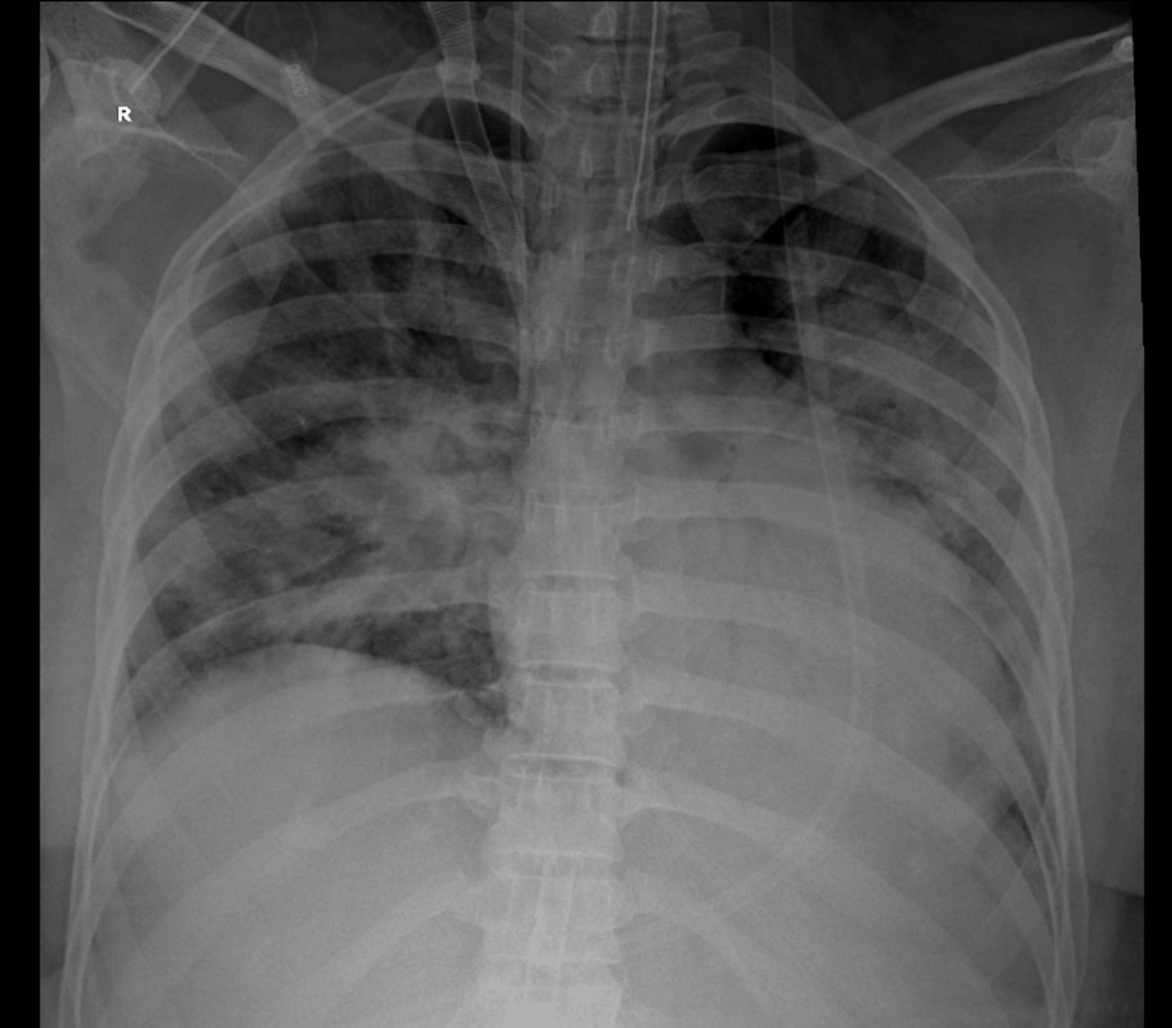
Progressive improvement on chest X-ray after ECMO initiation. ECMO, extracorporeal membrane oxygenation

On the following day, in view of worsening PaO₂/FiO₂ ratio, and a high Murray score of 3.8 and Sequential Organ Failure Assessment (SOFA) score of 14, early involvement of the CTVS and pulmonology teams enabled the timely initiation of ECMO. She was also started on empirical broad-spectrum antibiotics, including teicoplanin for methicillin-resistant Staphylococcus aureus (MRSA) coverage. A 25 Fr percutaneous femoral drainage cannula was inserted in the femoral vein, and a 22 Fr arterial return cannula was inserted in the right internal jugular vein (IJV). The position of the cannula was confirmed using transesophageal echocardiography (TEE). The drainage cannula was positioned in the hepatic inferior vena cava (IVC) just proximal to the hepatic vein, and the return cannula near the right atrium. Following this, she was initiated on venovenous ECMO (VV-ECMO) with flows of 2.5-2.9 L/minute. Over subsequent days, the patient showed progressive improvement on chest X-ray (Figure 3).

**Figure 4:**
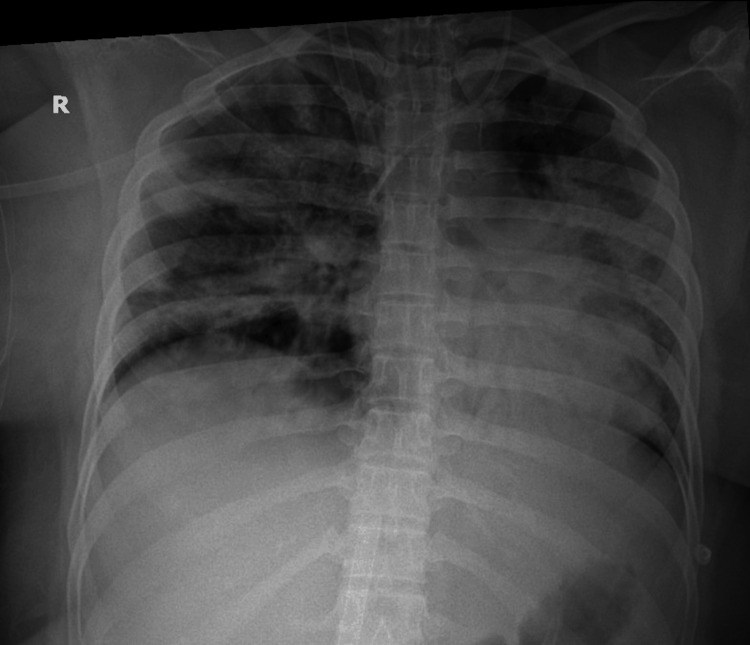
Worsening chest X-ray on day 9.

On day 9, a percutaneous tracheostomy was performed, and ventilatory and ECMO support were gradually reduced. Later that day, the patient developed a fever of 38 °C, for which a tracheostomy tube mini-bronchoalveolar lavage (TT-mini-BAL) was obtained. Following this, rapid desaturation necessitated higher FiO₂ and ECMO flows, followed by bronchoscopy in view of worsening chest X-ray (Figure 4).

**Figure 5:**
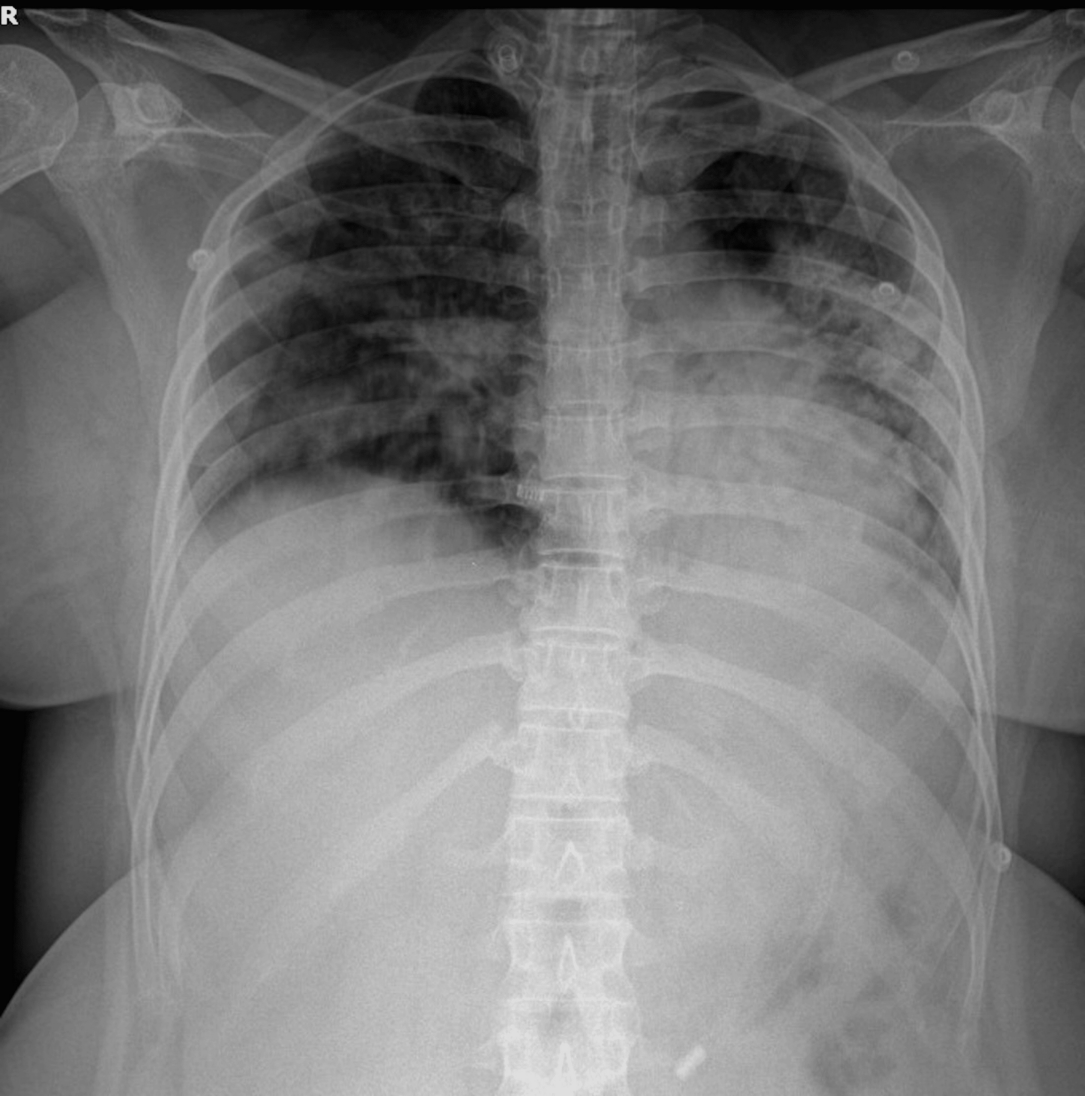
Chest X-ray on day 24.

TT-mini-BAL showed growth of multi-drug resistant Klebsiella, resistant to meropenem. Antibiotics were escalated to ceftazidime-avibactam and aztreonam following consultation with the infectious disease team. By day 18, repeat cultures revealed Acinetobacter baumannii sensitive to colistin, which was promptly initiated. Her clinical condition improved gradually, enabling weaning from ECMO, and she was decannulated after 17 days of support. On day 22, ventilator support was weaned and switched from synchronized intermittent mandatory ventilation (SIMV) to continuous positive airway pressure (CPAP), following which T-piece trials were given. As she was able to tolerate these satisfactorily, the tracheostomy tube was removed on day 24, with gradual reduction of supplemental oxygen. Serial chest X-rays showed progressive resolution of viral pneumonia and ARDS-related consolidation (Figure 5). Dietician and physiotherapy support contributed to recovery and functional rehabilitation.

She was transferred to the step-down ICU and later to the general medical ward on day 26. Table 1 provides a brief timeline of culture reports and antibiotic therapies administered. With steady improvement and good ambulation, she was discharged home on day 40. The favorable outcomes highlight the importance of early recognition and coordinated care in managing H1N1-associated ARDS in pregnancy.

**Table 1:** Antibiotic treatments and culture reports. TT mini-BAL, tracheostomy tube mini-bronchoalveolar lavage; ECMO, extracorporeal membrane oxygenation

Days since admission	Culture reports and antibiotic treatments
Day 1	Started on Inj Meropenem, Tab Azithromycin, and Inj Oseltamivir due to fever, elevated total blood cell counts, C-reactive protein (CRP), and procalcitonin. Cultures and sensitivity: • Blood: No bacterial growth. • Urine: No bacterial growth. • RT-PCR-H1N1 detected
Day 2	Started on Inj Teicoplanin for Methicillin-resistant Staphylococcus aureus (MRSA) coverage due to ECMO support and started on Cap. Doxycycline
Day 6	A repeat blood culture and sensitivity test revealed no bacterial growth. Cap Doxycycline was discontinued.
Day 8	Inj. Oseltamivir injections were stopped.
Day 12	The TT mini-BAL culture revealed the growth of multi-drug-resistant Klebsiella. Initiated on Inj Ceftazidime + Avibactam and Inj Aztreonam.
Day 18	TT-mini-BAL revealed growth of Acinetobacter baumannii and was started on Inj Colistin. Inj Ceftazidime + Avibactam and Inj Aztreonam were discontinued.
Day 23	Another TT-mini-BAL culture was sent, but no bacterial growth was observed.
Day 24	Inj Teicoplanin injections were discontinued.
Day 26	Inj Colistin treatment was discontinued.

## Discussion

This case highlights the importance of timely, multidisciplinary management of severe maternal H1N1 influenza infection with ARDS and ventilator-associated pneumonia (VAP). Early cesarean delivery was essential to safeguard fetal well-being and allow optimal maternal respiratory management, with antenatal corticosteroids supporting neonatal lung maturity.

Rapid confirmation of H1N1 influenza via reverse transcription-polymerase chain reaction (RT-PCR) enabled prompt initiation of antiviral therapy, which is safe in pregnancy [6]. Early antiviral administration has consistently been linked to reduced disease severity and improved outcomes [7], with current guidelines recommending initiation within 48 hours of symptom onset [8], likely contributing to improved maternal outcomes.

Following delivery, VV-ECMO was safely initiated to manage refractory hypoxemia in our patient, illustrating how timely obstetric intervention allowed the use of advanced supportive therapy. Careful coordination among the intensive care unit (ICU), obstetrics and gynecology, neonatology, pulmonology, and cardiothoracic teams was essential for recovery.

Secondary infections, including VAP caused by multidrug-resistant organisms, were promptly recognized and treated with targeted antimicrobial therapy, allowing gradual weaning from extracorporeal membrane oxygenation and mechanical ventilation.

This case also underscores the importance of preventive strategies, including influenza vaccination. Our patient had not received the H1N1 vaccine, which may have contributed to her susceptibility to severe infection. Evidence indicates that vaccination during pregnancy is safe and reduces the risk of complications, such as preterm birth and low birth weight [9,10].

Overall, this case underscores that coordinated multidisciplinary care, timely delivery, advanced postpartum respiratory support, prompt antiviral therapy, and vigilant infection management are key to achieving favorable maternal and neonatal outcomes.

## Conclusions

This case demonstrates that employing a comprehensive, multidisciplinary strategy, which encompasses early diagnosis, prompt antiviral therapy, timely delivery, and advanced supportive measures such as VV-ECMO, can result in favorable outcomes for both the mother and the infant in instances of severe H1N1-related ARDS during pregnancy. It also highlights the vital role of preventive vaccination and coordinated care in securing the best possible results for both mother and child.
